# Förderung der Zusammenarbeit von ÖGD und akademischen Einrichtungen in der Forschung: Erfahrungen aus dem BMG-geförderten Verbundforschungsprojekt Infektionsschutz.Neu.Gestalten (I.N.Ge)

**DOI:** 10.1055/a-2637-3372

**Published:** 2025-07-18

**Authors:** Anja Herrmann, Emily Piontkowski, Gottfried Roller, Stefan Brockmann, Fabian Nill, Kersten Wolfers, Susanne Minkwitz, Jana Hailer, Uwe Stengele, Peter Schäfer, Pierre Braun, Nadja Oster, Stefanie Joos, David Häske, Brigitte Joggerst, Monika A Rieger

**Affiliations:** 1 Zentrum für öffentliches Gesundheitswesen und Versorgungsforschung, Universitätsklinikum Tübingen, Tübingen, Germany; 2 Landesgesundheitsamt Baden-Württemberg, Ministerium für Soziales, Gesundheit und Integration Baden-Württemberg, Stuttgart, Germany; 3 Kreisgesundheitsamt Reutlingen, Reutlingen, Germany; 4 Gesundheitsamt Enzkreis, Pforzheim, Germany; 5 Gesundheitsamt, Stadt Mannheim, Mannheim, Germany; 6 Institut für Allgemeinmedizin und interprofessionelle Versorgung, Universitätsklinikum Tübingen, Tübingen, Germany; 7 Gesundheitsamt, Stadt Karlsruhe, Karlsruhe, Germany; 8 Institut für Arbeitsmedizin, Sozialmedizin und Versorgungsforschung, Universitätsklinikum Tübingen, Tübingen, Germany

**Keywords:** ÖGD, Kooperation, Forschung, Praxis, Evidenz, Netzwerk, public health service, cooperation, research, practice, evidence, network

## Abstract

Das Leitbild des ÖGD von 2018 betont die Bedeutung der Wissenschaftlichkeit und die Zusammenarbeit mit wissenschaftlichen Einrichtungen. 2020 griff das Bundesministerium für Gesundheit (BMG) diesen Aspekt in einer Förderausschreibung zur Stärkung der Kooperation zwischen ÖGD und Public-Health-Forschung auf. Im Rahmen dieser Ausschreibung entstand der Forschungsverbund Infektionsschutz.Neu.Gestalten (I.N.Ge) mit vier Gesundheitsämtern und einer Universität (Förderzeitraum: 09/2021–08/2024). Ziel war, die Arbeit des ÖGD in Gesundheits- und Infektionsschutz evidenzbasiert zu verbessern. Am Beispiel des Infektionsschutzes in der COVID-19-Pandemie wurden Digitalisierung, Qualitätssicherung, Risikokommunikation und besondere Personengruppen untersucht. Dabei kam ein partizipativer, transformativer Ansatz mit Reallaboren und -Experimenten zum Einsatz. Aus der intensiven Kooperation wurden „Lessons Learned für eine erfolgreiche Zusammenarbeit zwischen ÖGD und universitären Einrichtungen abgeleitet. Wichtige Faktoren sind passende Rahmenbedingungen, gezielte Ressourcenplanung und klare Rollendefinitionen. Themenfelder wurden identifiziert, welche künftige Kooperationen effektiver und nachhaltiger gestalten können. Die daraus entwickelten Handlungsempfehlungen sollen u. a. die Konzeption gemeinsamer Forschungsprojekte unterstützen. I.N.Ge zeigt, dass es für eine stärkere Zusammenarbeit verbesserter Rahmenbedingungen bedarf. Förderprojekte sollten flexible Kooperationsmodelle ermöglichen, die sich an spezifische Ressourcen und Kompetenzen der Partner anpassen. Eine vorausschauende Ressourcenplanung ist essenziell – inklusive Stellenkalkulationen, Vertretungsregelungen und zusätzlicher Kapazitäten für Einarbeitung oder methodische Anpassungen. Klare Rollendefinitionen und Zuständigkeiten sollten bereits in der Ausschreibungsphase festgelegt werden, um Transparenz zu schaffen und die Umsetzung zu optimieren. I.N.Ge verdeutlicht, dass die Zusammenarbeit zwischen ÖGD und akademischen Einrichtungen gefördert werden muss, um den ÖGD weiterzuentwickeln und zu stärken.

## Abstract (en)

The 2018 mission statement of the ÖGD emphasises the importance of scientific research and cooperation with scientific institutions. In 2020, the Federal Ministry of Health (BMG) took up this aspect in a call for proposals to strengthen cooperation between the ÖGD and public health research. In the context of this call, the research network 'Infektionsschutz.Neu.Gestalten (I.N.Ge)' was established with four health authorities and one university (funding period: 09/2021–08/2024). The aim was to improve the evidence-based work of the ÖGD in the field of health and infection protection. Digitalisation, quality assurance, risk communication and special groups were examined using the example of infection control in the COVID-19 pandemic. A participatory, transformative approach with real-world laboratories and experiments was used. Lessons for successful cooperation between public health and university institutions were drawn from the intensive collaboration. Important were appropriate frameworks, targeted resource planning and clear role definitions. Issues were identified that could make future collaboration more effective and sustainable. The resulting recommendations for action are intended, among other things, to support the conception of joint research projects. I.N.Ge showed that improved framework conditions were necessary for increased cooperation. Funding projects should allow for flexible cooperation models that adapt to the specific resources and competencies of the partners. Forward-looking resource planning was determined to be essential – including job calculations, substitution arrangements and additional capacity for induction or methodological adaptation. Clear roles and responsibilities should be defined at the tendering stage to create transparency and optimise implementation. I.N.Ge emphasized the need to promote cooperation between the ÖGD and academic institutions in order to further develop and strengthen the public health service. I.N.Ge emphasises that cooperation between the ÖGD and academic institutions must be encouraged in order to further develop and strengthen the ÖGD.

## Einleitung

Die COVID-19-Pandemie hat die Bedeutung des Öffentlichen Gesundheitsdienstes (ÖGD) hervorgehoben. Trotz des großen Engagements des ÖGD in dieser Krisensituation bleibt eine inhaltliche und strukturelle Weiterentwicklung unabdingbar. Neben ausreichend personellen Ressourcen bedarf es belastbarer Strukturen für den Transfer von gesichertem Wissen sowie geeigneter Umsetzungsmöglichkeiten für evidenzbasierte, innovative Maßnahmen. Die Erfahrungen aus der Pandemie verdeutlichen, dass Verbesserungen in den Kernbereichen und Prozessen des ÖGD notwendig sind. Dazu gehören insbesondere die Digitalisierung, die Qualitätssicherung, die Risikokommunikation sowie die Berücksichtigung der Bedürfnisse spezifischer Bevölkerungsgruppen.

Das Verbundprojekt „Infektionsschutz.Neu.Gestalten (I.N.Ge)" wurde ins Leben gerufen, um die Kooperation zwischen Wissenschaft und ÖGD zu fördern und weiterzuentwickeln. Die zentrale Fragestellung des Projekts lautete, wie der ÖGD – insbesondere im Gesundheits- und Infektionsschutz – evidenzbasiert und nachhaltig gestärkt werden könne. Dafür wurde eine transdisziplinäre, transformative Forschungsmethodik auf Basis von Reallaboren gewählt. Die Reallabore befassten sich zum einen mit der Zusammenarbeit der beteiligten Einrichtungen und zum anderen mit den inhaltlich ausgerichteten Themen. Drei Gesundheitsämter und das Landesgesundheitsamt in Baden-Württemberg (Enzkreis, Mannheim, Reutlingen und Landesgesundheitsamt BaWü) sowie das Zentrum für Öffentliches Gesundheitswesen und Versorgungsforschung (ZÖGV), Universitätsklinikum Tübingen arbeiteten an den inhaltlichen Themenschwerpunkten Digitalisierung, Risikokommunikation, Qualitätssicherung sowie an Konzepten für Gruppen mit besonderen Bedarfen.

Zu den oben benannten Themenfeldern wurden in den vier Reallaboren spezifische Forschungsfragen in partizipativer Zusammenarbeit entwickelt, Interventionen erprobt, angepasst und mit qualitativen sowie quantitativen Methoden evaluiert. Die Reallaborplattform diente als zentraler Knotenpunkt für den Austausch und Transfer zwischen den Projektpartnerinnen und Projektpartnern und unterstützte die Verstetigung der gewonnenen Erkenntnisse. Ergänzend bietet das ZÖGV bedarfsorientierte Methodentrainings an, als Fortbildung zur Förderung wissenschaftlicher Methodenkompetenz. Das Verbundprojekt I.N.Ge liefert innovative Ansätze für eine nachhaltige evidenzbasierte Weiterentwicklung des ÖGD.

Forschungskooperationen schaffen Synergien zwischen Praxis und Forschung und ermöglichen die Etablierung neuer Standards für die Bewältigung von Gesundheitskrisen. Die im Forschungsverbund gewonnenen Erkenntnisse ermöglichen praxisorientierte Handlungsempfehlungen, die sowohl auf politischer als auch operativer Ebene zu einer langfristigen Stärkung des ÖGD beitragen können.

Aus dem Forschungsverbund I.N.Ge sind eine Reihe wissenschaftlicher Originalarbeiten hervorgegangen bzw. gehen daraus hervor, die sich mit Methodenkompetenz im ÖGD sowie wissenschaftlichen Kompetenzen in der Versorgungsforschung am Universitätsklinikum befassen 1 2 . Darüber hinaus haben zahlreiche Kongressbeiträge erste Erkenntnisse aus dem Verbund präsentiert 3 4 5 6 7 8 9 , insbesondere zu Themen wie Risikokommunikation 10 , digitalen Kompetenzen von ÖGD-Mitarbeitenden 11 und der Prävention sexuell übertragbarer Infektionen 12 . Diese Beiträge leisten einen wichtigen Beitrag zur empirischen Absicherung der gewonnenen Erkenntnisse und unterstützen damit die Entwicklung von belastbarer Evidenz für die Praxis.

Das vorliegende Policy Paper widmet sich den „Lessons Learned“ aus dem Forschungsverbund I.N.Ge. Es beleuchtet die Schlüsselfaktoren für erfolgreiche Forschungskooperationen, darunter die Rahmenbedingungen, Ressourcenplanung, Rollendefinition und strukturellen Voraussetzungen, die es für eine effektive Zusammenarbeit zwischen ÖGD und Forschung bedarf. Die daraus resultierenden Erkenntnisse sind nicht nur für den wissenschaftlichen Diskurs, sondern auch für die politische und administrative Praxis von Relevanz und werden hier dargestellt.

## Methodik

Die Lessons Learned wurden partizipativ durch alle Beteiligten des Forschungsverbunds I.N.Ge identifiziert. Zum einen wurde auf der operativen Verbund-Ebene wie auch intern in den beteiligten Institutionen relevante Aspekte zusammengetragen und daraus Themenfelder für ein World Café eingebracht. Die Projektbeteiligten aus Leitungs- und Mitarbeitenden-Ebene reflektierten, diskutierten und bewerteten die einzelnen Aspekte. Mit Hilfe der qualitativen Inhaltsanalyse wurden die erhobenen Erkenntnisse transkribiert und ausgewertet und eine Zuordnung in Haupt- und Subkategorien vorgenommen.

## Erfahrungen aus dem Verbundprojekt

### Projektinhalte und Verantwortlichkeiten jeweils entsprechend der Interessen und Möglichkeiten aller beteiligten Einrichtungen konzipieren!

Bereits in der Phase der Antragserstellung ist eine gründliche Klärung der Interessen und Möglichkeiten der einzelnen Kooperationspartner erforderlich. Dies umfasst sowohl inhaltliche, methodische Interessen und Kompetenzen als auch die Möglichkeiten im Rahmen des geplanten Projektes (z. B. Personalausstattung, zeitgleich zu bearbeitende andere Aufgaben). Diese grundsätzliche Offenheit für eine von Projekt zu Projekt unterschiedlich gestaltete Zusammenarbeit mehrerer ÖGD-Partner untereinander oder auch mit einer universitären Einrichtung kann beispielsweise dazu führen, dass einzelne Partner (aus dem ÖGD oder universitär) projektspezifisch unterschiedliche Aufgaben übernehmen – sei es die „vollständige eigenständige Bearbeitung eines wissenschaftlichen (Sub-)Projektes“ oder die Beteiligung an einzelnen Arbeitsschritten in (Sub-)Projekten unter Federführung anderer Partner*, z. B. „Ermöglichen des Feldzugangs für Datenerhebung“ oder „Beteiligung an Instrumentenentwicklung“. Ausschreibungen und Projektförderungen sollten diese Offenheit ermöglichen und eine Reflexion der jeweils spezifischen Projektkonzeption anregen.

Bezüglich des Aufbaus und der Etablierung von (Forschungs-) Gesundheitsämtern, die sich in der Gestaltung der Forschungslandschaft einbringen oder künftig mehr einbringen möchten, ist diese Flexibilität in der Ausgestaltung sehr relevant. Es braucht Spielräume und freie Entscheidungsmöglichkeiten, die auf Grundlage der individuellen Ressourcen jeweils projektspezifisch im Gesundheitsamt getroffen werden können.

### Was sollte bei der Ressourcenplanung berücksichtigt werden?

Unter Berücksichtigung des Vorhabens sollten verfügbare Personalressourcen hinsichtlich Kompetenzen, Qualifikation und möglichem Stellenumfang genau betrachtet werden. Möglicherweise im zeitlichen Verlauf erforderliche Vertretungen einzelner Mitarbeitenden (durch z. B. Eltern- oder Pflegezeit) sollten – soweit möglich – zusätzlich berücksichtigt und eingeplant werden. Stellenanteile sollten großzügig kalkuliert werden, da insbesondere für praxisorientierte oder gar transdisziplinäre Projekte bzw. die Verwendung innovativer Methoden immer mit einem erhöhten Arbeitsvolumen zu rechnen ist, bspw. durch Einarbeitungszeiten oder Zeiten für die Etablierung eines Netzwerks. Sind im Team bestimmte erforderliche Expertisen noch nicht vorhanden, sollte die Ressourcenplanung Zeiten für den Aufbau dieser Kompetenzen im Team vorsehen. Für die Zusammenarbeit verschiedener Einrichtungen, zumal wenn diese jeweils einen unterschiedlichen Tätigkeitsschwerpunkt haben (z. B. primär Aufgaben des ÖGD oder primär Forschung) ist mindestens für die erste Kooperation ausreichend Zeit für die Vernetzung und das Kennenlernen der jeweiligen Rahmenbedingungen für die gemeinsame Projektarbeit einzuplanen (z. B. durch Hospitationen). Dies ist bei der Finanzplanung zu berücksichtigen. Längerfristig angelegte Austauschformate oder gar Abordnungsstellen können das nachhaltige Gelingen von Forschungskooperationen unterstützen.

### Besonderheiten wissenschaftlichen Arbeitens in der Forschung anerkennen

Wissenschaftliches Arbeiten im Rahmen von Forschungsprojekten unterliegt anderen Rahmenbedingungen als Projekte, die z. B. in Erfüllung eines gesetzlich begründeten Auftrags mit vergleichbaren wissenschaftlichen Methoden bearbeitet werden. Dies gilt insbesondere für die Erfordernis einer berufsrechtlichen Beratung durch die zuständige Ethik-Kommission auf der Basis eines hierfür entwickelten Studienprotokolls, das gängige Richt- und Leitlinien berücksichtig (z. B. DFG-Leitlinien guter wissenschaftlicher Praxis, WMA Deklaration von Helsinki). Ebenso ist das etwaige Erfordernis einer Beratung durch die zuständigen Datenschutzbeauftragten bzgl. des Datenschutzkonzeptes zu berücksichtigen, an das bei Forschungsprojekten teilweise andere Anforderungen gestellt werden als bei z. B. Projekten im Bereich der Qualitätssicherung.

Führen ÖGD-Partner federführend oder selbständig Datenerhebungen im Rahmen von Forschungsprojekten durch, ist in der Regel die Ethik-Kommission der jeweiligen Landesärztekammer zuständig. Bei Kooperationsprojekten muss vorab geklärt werden, welche Ethik-Kommission zuerst um die berufsrechtliche Beratung in Bezug auf das Studienprotokoll gebeten wird und welche Ethik-Kommission(en) in der Folge angesprochen werden. Entsprechende zeitliche Aufwände bis hin zu Gebühren der Ethik-Kommissionen sind in der Ressourcenplanung zu berücksichtigen. Wissenschaftliche Veröffentlichungen zur Methodik und den Ergebnissen von Forschungsprojekten sind unabdingbar für den Forschungsdiskurs. Bei Veröffentlichungen sollten gängige Empfehlungen (z. B. passende Reporting Guidelines, Empfehlungen des International Committee of Medical Journal Editors (ICMJE)) berücksichtigt werden. Das Erstellen wissenschaftlicher Veröffentlichungen muss in der Zeit- und Ressourcenplanung berücksichtigt werden.

### Welche strukturellen Voraussetzungen sollten gegeben sein?

Für alle Aspekte der Forschungsorganisation braucht es Expertise und Erfahrung. Administrative Prozesse, auch im Hinblick auf die Verwaltung von Drittmitteln, die ggf. notwendige Einbindung des Personalrats im Fall von Befragungen der Mitarbeitenden oder weiteren Stabstellen, sollten bei allen beteiligten Projektpartnerinnen und Projektpartnern mitgedacht und mit einem zeitlichen Mehraufwand geplant werden.

Voraussetzungen für evidenzbasiertes, wissenschaftliches Arbeiten (z. B. Zugriff auf Literaturdatenbanken, Software für Datenerhebung und Auswertung) sollten gegeben sein oder in der Budget- oder Kooperationsplanung berücksichtigt werden. Technische Voraussetzungen sollten bei allen Partnerinnen und Projektpartnern gegeben sein. Beschaffungsanträge können zeitlich lange dauern, was vorab bei der Finanzplanung berücksichtigt werden sollte.

Eine wissenschaftliche Begleitung aus dem universitären Setting kann den Aufbau von Forschungsexpertise in ÖGD-Einrichtungen wesentlich unterstützen. Niederschwellige Angebote z. B. in Form von Methodenprogrammen aus der Versorgungsforschung fördern die Kompetenzen der Teilnehmenden in diesem Bereich und bauen neue Expertise auf. Personal aus dem ÖGD mit einem entsprechenden Aufgabenspektrum sollte die Möglichkeit haben, an entsprechenden Fort- und Weiterbildungsangeboten teilnehmen zu können. Auch dieser Aspekt sollte bei der Budgetplanung Berücksichtigung finden.

### Wie kann man unterschiedliche Organisationskulturen kennen- und verstehen lernen?

Jede Institution lebt und arbeitet nach der eigenen Organisationskultur. In Kooperationsprojekten und der Vorbereitung dieser ist es unerlässlich, sich Zeit für das gegenseitige Kennenlernen zu nehmen, idealerweise in wiederholten persönlichen Begegnungen. Eine transparente Kommunikation sowie die Klärung von Rollen und Verantwortlichkeiten sind ebenfalls von grundlegender Bedeutung für eine gelingende Zusammenarbeit.

So können die jeweils andere Organisationskultur und Aufgabenbereiche mit den damit einhergehenden Tätigkeiten und Anforderungen verstanden und verschiedenen Kulturen projektbezogen einander angenähert werden.

Seitens der Führungsebene ist es wichtig, entsprechende Forschungsprojekte insbesondere im Rahmen von Kooperationen mit anderen Institutionen unterstützend und mit einem hohen Engagement zu begleiten. Landräte und Landrätinnen bzw. Oberbürgermeisterinnen und Oberbürgermeister sollten zumindest bei allen Projekten mit (möglichem) Praxisbezug ausreichend informiert und eingebunden werden.

In den Gesundheitsämtern werden bereits wissenschaftliche Methoden angewandt, bspw. in der Gesundheitsberichterstattung oder im Infektionsschutz. Gleichwohl gibt es ein großes Interesse auf Seiten des ÖGD, stärker im Bereich der Wirksamkeit der von ihnen durchgeführten Maßnahmen aktiv zu werden und damit auch komplexere Datenerhebungen und -analysen durchzuführen. Dies auch im Hinblick auf eine stärkere Analyse von Kosten-Nutzen-Verhältnissen der Maßnahmen vor dem Hintergrund von schwindenden personellen und finanziellen Ressourcen.

Die konsequentere Nutzung wissenschaftlicher Instrumente für Wirksamkeitsnachweise und Kosten-Nutzen-(Effizienz-)Analysen würde sehr unterstützt durch eine gesetzliche Verankerung dieser Aufgabe im ÖGDG. Daraus entstünde ein klarer Auftrag und ein Rückhalt für den ÖGD in Kooperation mit wissenschaftlichen Einrichtungen wissenschaftliche Fragestellungen auch mit eigenen Ressourcen zu bearbeiten.

#### Handlungsempfehlungen von der Forschungspraxis in die Politik

Zur Förderung der Zusammenarbeit zwischen dem ÖGD und wissenschaftlichen Einrichtungen in der Forschung können folgende Handlungsempfehlungen für politische Entscheidungsträger*innen abgeleitet werden:

##### Flexibilität in der Gestaltung von Förderprogrammen

Bereits bei der Konzeption und Ausschreibung von Förderprojekten sollten unterschiedliche Kooperationsmodelle sowie flexible Rollenverteilungen explizit berücksichtigt werden. Die Förderprogramme sollen es ermöglichen, dass Projektpartner ihre spezifischen Ressourcen und Kompetenzen optimal in das Projekt einbringen können. Dies beinhaltet die Option, dass einzelne Partner eigenständig Teilprojekte bearbeiten oder gezielte Unterstützung bei spezifischen Arbeitsschritten leisten. Diese Flexibilität gewährleistet eine bedarfsgerechte und effektive Zusammenarbeit.

##### Ressourcenplanung mit Weitblick

Eine angemessene und vorausschauende Ressourcenplanung ist essenziell für den Erfolg transdisziplinärer Projekte. Die Politik kann dabei sicherstellen, dass Förderprogramme eine dem Projekt angemessene Kalkulation von Stellenanteilen sowie ausreichende Mittel für Vertretungsregelungen und die Qualifizierung von Mitarbeitenden vorsehen. Dabei müssen auch der zusätzliche Aufwand durch Einarbeitungszeiten, die Integration neuer Methoden sowie die Kapazitäten für im Hintergrund agierende Mitarbeitende berücksichtigt werden. Diese umfassende Planung erhöht die Effizienz und Qualität der Projektarbeit.

##### Klare Rollendefinition und Verantwortlichkeiten

Förderprogramme sollten Anreize schaffen, bereits in der Planungs- und Ausschreibungsphase klare Rollen und Zuständigkeiten zu definieren. Eine präzise Rollenverteilung zwischen den Projektpartnern fördert die Transparenz und Verbindlichkeit in der Zusammenarbeit. Zudem trägt sie zu einem optimierten Arbeitsprozess bei, da Verantwortlichkeiten von Beginn an klar geregelt und Aufgaben spezifisch auf die jeweiligen Partner abgestimmt sind.

##### Förderung transdisziplinärer Ansätze

Die Politik sollte transdisziplinäre Projekte durch gezielte Förderprogramme unterstützen. Diese Projekte zeichnen sich durch komplexe Anforderungen an Vernetzung, innovativer Methodik und längerfristige Einarbeitungszeiten aus, die eine umfangreiche Ressourcenausstattung erforderlich machen. Die langfristige Bereitstellung finanzieller Mittel ist notwendig, um den Aufbau neuer Kompetenzen und die intensive Zusammenarbeit der Akteure zu ermöglichen.

##### Stärkung der Infrastruktur

Die Bereitstellung von Mitteln für technische Infrastruktur, wie Software zur Datenerhebung und -auswertung, sowie für den Zugang zu Literaturdatenbanken ist unabdingbar. Diese Ressourcen sind eine Grundvoraussetzung für evidenzbasierte und wissenschaftliche Arbeitsweisen, die sowohl in der Forschung als auch in der Praxis Anwendung finden.

##### Förderung von Weiterbildung und Kompetenzaufbau

Die Politik sollte projektbezogene Fort- und Weiterbildungsprogramme fördern, die den Mitarbeitenden im ÖGD den Erwerb neuer Kompetenzen ermöglichen. Dies umfasst auch Hospitationen und andere Vernetzungsaktivitäten, die den Austausch zwischen den Projektpartnern erleichtern. Der Aufbau von Expertise und die Weiterentwicklung der Mitarbeitenden ist entscheidend für die Qualität und Nachhaltigkeit von Kooperationen.

## Schlussfolgerung

Die im Forschungsverbund Infektionsschutz.Neu.Gestalten (I.N.Ge) gewonnenen Erkenntnisse zeigen deutlich, wie wichtig eine enge und strukturierte Zusammenarbeit zwischen dem Öffentlichen Gesundheitsdienst (ÖGD) und wissenschaftlichen Einrichtungen für die Weiterentwicklung der Public-Health-Forschung ist. Diese Kooperationen ermöglichen nicht nur eine praxisnahe Forschung, sondern leisten auch einen Beitrag zur Stärkung des ÖGD als bedeutsamer Akteur im Gesundheitssystem.

Die Erkenntnisse aus I.N.Ge zeigen, dass die Zusammenarbeit von ÖGD und akademischen Einrichtungen in der Forschung besonderer Rahmenbedingungen bedarf.

Die formulierten Handlungsempfehlungen verdeutlichen, wie die Politik diese Zusammenarbeit langfristig und nachhaltig fördern kann. Die Bereitstellung von flexiblen und ausreichenden Ressourcen, die Förderung transdisziplinärer Ansätze sowie der Aufbau von Kompetenzen im ÖGD sind essenziell, um Forschung und Praxis effektiv miteinander zu verknüpfen. Darüber hinaus ist die Verankerung wissenschaftlicher Methoden im gesetzlichen Auftrag des ÖGD ein zentraler Schritt, um evidenzbasierte Arbeitsweisen in den Routinetätigkeiten zu stärken und die Qualität der Gesundheitsversorgung zu verbessern.

Um die identifizierten Herausforderungen zu bewältigen, bedarf es eines kontinuierlichen Austauschs zwischen Wissenschaft, Praxis und Politik. Maßnahmen wie die Schaffung von (Forschungs-)Gesundheitsämtern, klare Rollendefinitionen in Projekten und eine stärkere Einbindung kommunaler Führungsebenen können dazu beitragen, die Zusammenarbeit effizienter und zielführender zu gestalten.

I.N.Ge zeigt, dass die Förderung der Zusammenarbeit von ÖGD und akademischen Einrichtungen wichtig ist, um den ÖGD weiterzuentwickeln und zu stärken.

Diese Partnerschaft kann erheblich dazu beitragen, die Gesundheit der Bevölkerung zu verbessern und die Resilienz des ÖGD gegenüber zukünftigen Herausforderungen zu stärken.

## Fördermittel

Bundesministerium für Gesundheit — http://dx.doi.org/10.13039/501100003107; ZMI1-2521FSB111

Das Institut für Arbeitsmedizin, Sozialmedizin und Versorgungsforschung, Universitätsklinikum Tübingen, erhält eine institutionelle Förderung durch den Verband der Metall- und Elektroindustrie Baden-Württemberg e.V. (Südwestmetall)
